# A marker of biological ageing predicts adult risk preference in European starlings, *Sturnus vulgaris*

**DOI:** 10.1093/beheco/ary009

**Published:** 2018-02-24

**Authors:** Clare Andrews, Daniel Nettle, Sophie Reichert, Tom Bedford, Pat Monaghan, Melissa Bateson

**Affiliations:** 1 Centre for Behaviour and Evolution, Institute of Neuroscience and Newcastle University Institute of Ageing, Henry Wellcome Building, Newcastle University, Framlington Place, Newcastle upon Tyne, UK; 2 Institute of Biodiversity, Animal Health and Comparative Medicine, Graham Kerr Building, University of Glasgow, Glasgow, UK; 3 Department of Animal and Plant Sciences, University of Sheffield, Alfred Denny Building, Western Bank, Sheffield, UK

**Keywords:** ageing, developmental plasticity, early life adversity, life expectancy, risk sensitive foraging, risk taking, starling, telomere

## Abstract

Why are some individuals more prone to gamble than others? Animals often show preferences between 2 foraging options with the same mean reward but different degrees of variability in the reward, and such risk preferences vary between individuals. Previous attempts to explain variation in risk preference have focused on energy budgets, but with limited empirical support. Here, we consider whether biological ageing, which affects mortality and residual reproductive value, predicts risk preference. We studied a cohort of European starlings (*Sturnus vulgaris*) in which we had previously measured developmental erythrocyte telomere attrition, an established integrative biomarker of biological ageing. We measured the adult birds’ preferences when choosing between a fixed amount of food and a variable amount with an equal mean. After controlling for change in body weight during the experiment (a proxy for energy budget), we found that birds that had undergone greater developmental telomere attrition were more risk averse as adults than were those whose telomeres had shortened less as nestlings. Developmental telomere attrition was a better predictor of adult risk preference than either juvenile telomere length or early-life food supply and begging effort. Our longitudinal study thus demonstrates that biological ageing, as measured via developmental telomere attrition, is an important source of lasting differences in adult risk preferences.

## Teaser

Why do some individuals gamble more than others? Existing theories, based on how close animals are to starvation, have been only weakly supported by evidence. We found that faster ageing birds were less likely to gamble while foraging. We measured biological ageing in starlings from the shortening of DNA sequences called telomeres. Birds’ whose telomeres shortened more had stronger preferences for a foraging option yielding a consistent amount of food over a variable amount.

## INTRODUCTION

Decision-making under risk is an important topic in disciplines as diverse as psychology, economics, anthropology, and biology (Hintze et al. 2015; Lim et al. 2015). Like humans, wild animals face the fact that most actions do not have a single predictable outcome but a range of possible consequences. Variable outcomes are prevalent in foraging decisions because food resources vary in space and time. Experiments on a range of species have shown that individual foragers demonstrate consistent preferences between alternative foraging options yielding a constant “safe” return versus a variable “risky” return, despite both yielding the same mean return rate (Kacelnik and Bateson 1996; Kacelnik and Bateson 1997; Bateson 2002). Previous attempts to explain this variation in risk preference have predominantly focused on an animal’s energy budget, that is, whether energy intake is sufficient to meet the animal’s metabolic requirements and hence maintain body weight (reviewed in Bateson 2002; Kacelnik and El Mouden 2013). The classic “energy budget rule” (Stephens 1981) predicts that an animal on a positive energy budget should be risk averse, whereas an animal on a negative energy budget should be risk prone (Caraco et al. 1980; Stephens 1981). However, an extensive literature testing the energy budget rule offers only weak support (Brito e Abreu and Kacelnik 1995; Kacelnik and Bateson 1997; Bateson and Kacelnik 1998; Weber et al. 2004; Kacelnik and El Mouden 2013). Alternative sources of variation in risk preference beyond energy budgets therefore deserve attention. Here, we consider a potential role for biological age.

Optimality models of risk sensitive foraging predict shifts between risk-prone and risk-averse foraging according to an animal’s energy budget (Stephens 1981; Stephens and Krebs 1986; McNamara and Houston 1992), because energy budget alters the shape of the relationship between the amount of food gained and evolutionary fitness (Kacelnik and Bateson 1997). Any nonlinear relationship between energy intake and fitness should result in risk-sensitive foraging decisions (McNamara et al. 1991; Houston et al. 2014). The shape of this fitness function could be influenced by factors besides energy budget (Houston et al. 2014). One such possibility is an animal’s rate of ageing, since faster-ageing individuals would have reduced life expectancy and residual reproductive value (the expected future number of offspring produced over the remainder of the lifetime, as a function of the animals’ current state; Houston and McNamara 1999). Houston et al. (2014) highlight that an animal’s willingness to take foraging risks should be affected by its residual reproductive value (the direction depending on the shape of the underlying fitness function relating energy reserves to reproductive value). McNamara et al. (1991; see also Merad and McNamara 1994) modeled the influence of mortality rate (which is the inverse of life expectancy) on risk-sensitive foraging. They showed that as life expectancy decreases, the range of circumstances under which it is optimal to be risk prone increases. Furthermore, as reserves increase, life expectancy becomes relatively more important, such that decreasing life expectancy promotes risk-prone foraging in animals that are at low risk of starving to death (McNamara et al. 1991). We therefore predicted that accelerated biological ageing (i.e., lowered life expectancy, hence higher mortality) would increase an animal’s propensity for risk-prone foraging.

The rate of biological ageing varies among individuals (Belsky et al. 2015) and can be objectively assessed by measuring biomarkers that typically change with chronological age, but that provide a better prediction of life expectancy than chronological age itself (Levine 2013; Dontsov and Krut’ko 2015). Telomere length has emerged as a candidate cellular biomarker of biological age (Epel 2009). Telomeres are repetitive DNA sequences forming protective “caps” on eukaryotic chromosomes that shorten with chronological age (Hastie et al. 1990; Epel 2009; Heidinger et al. 2012), a process accelerated by various forms of stress in a range of species including humans and birds (Nettle et al. 2013; Boonekamp et al. 2014; Hau et al. 2015; Nettle, Monaghan, et al. 2015; Bateson 2016; Nettle, Andrews, Reichert, et al. 2017). Both telomere length and developmental telomere attrition have been shown to predict longevity and reproductive success in birds (Pauliny et al. 2006; Kimura et al. 2008; Monaghan 2010; Heidinger et al. 2012; Boonekamp et al. 2014; Asghar et al. 2015), with some evidence suggesting that telomere attrition is the better predictor (Boonekamp et al. 2014).

Here, we test for the first time the prediction that biological ageing, measured by developmental telomere attrition, increases risk-prone foraging in European starlings (*Sturnus vulgaris*), a bird species extensively studied in the context of risk-sensitive foraging decisions (Reboreda and Kacelnik 1991; Bateson and Kacelnik 1995; Brito e Abreu and Kacelnik 1995). Our previous work in starlings has demonstrated associations between developmental telomere attrition and other aspects of foraging decision-making, including judgment of ambiguous stimuli associated with food (Bateson, Emmerson, et al. 2015), impulsivity for immediate over delayed food rewards (Bateson, Brilot, et al. 2015), and foraging motivation (as measured by the breakpoint on progressive ratio schedules; Nettle, Andrews, et al. 2015). In the present experiment, we measured the risk preference of starlings presented with repeated choices between fixed and variable amounts of food with equal mean amount. We studied a cohort of hand-reared birds whose developmental histories, including their developmental telomere attrition rates, were known in detail. As reported elsewhere (Nettle, Andrews, Reichert, et al. 2017), as nestlings we had experimentally controlled the birds’ food supply and their required begging effort. These factors contributed to the observed variation in developmental telomere attrition (Nettle, Andrews, Reichert, et al. 2017). Since developmental telomere attrition integrates multiple sources of developmental influence on biological ageing (Bateson 2016), we expected that developmental telomere attrition would be a stronger predictor of adult risk preference than the early-life environmental parameters which we had experimentally manipulated.

## METHODS

### Ethics

Our study adhered to the ASAB/ABS Guidelines for the ethical treatment of animals, was approved by Newcastle University local ethical review committee and was conducted under UK Home Office project licence (numbers PPL 60/4073 and 70/8089) and Natural England license (number 20121066); see Electronic Supplementary Materials for further details.

### Study animals, developmental manipulation, and telomere attrition

Subjects were 32 European starlings (16 male, 16 female; sexed molecularly after assignment to developmental treatments; see Nettle, Andrews, Reichert, et al. 2017) from 8 natal families belonging to a cohort of chicks hatched in the wild in May 2014 in a nest-box population on farms in Northumberland, UK. The birds were subject to a developmental manipulation described in full elsewhere (Nettle, Andrews, Reichert, et al. 2017). Briefly, on posthatching day 5, quartets of siblings were brought to the lab where they were hand reared. On day 6 and continuing until day 15, we simultaneously manipulated amount of food (hereafter Amount: Plenty or Lean) and the begging effort (Effort: Easy or Hard) experienced by the nestlings, in a 2 × 2 factorial design. Nestlings allocated to the Plenty groups were fed ad libitum to satiation at each feeding visit, while the Lean groups received a proportion of the amount consumed by the corresponding Plenty group (approximately 73%, although this was varied from visit to visit in order to replicate growth trajectories of the slowest-growing chicks in wild nests). To manipulate begging effort, nestlings in the Hard groups received twice as many visits as those in the Easy groups, but were fed during only half of those visits while in the other half they were stimulated to beg for 2 min without receiving food. From day 16 onwards, all birds received ad libitum food. The manipulation affected growth rates, body weight, and skeletal size at the time of fledging (Nettle, Andrews, Reichert, et al. 2017). Once the fledglings became independent (approximately 4 weeks posthatch), they were transferred into mixed-sex, mixed-treatment groups to 2 indoor aviaries (215 × 340 cm and 220 cm high; ca. 18 °C; 40% humidity; 15:9 h light:dark cycle) and were fed ad libitum on domestic chick crumb (Special Diets Services “Poultry Starter [HPS]”), supplemented with cat biscuits (Royal Canin Ltd.), dried insect food (Orlux insect pâté), live mealworms, and fruit.

We measured the attrition of erythrocyte telomeres over the course of the manipulation and in the juvenile period immediately following it. Telomere length was measured via qPCR from blood samples taken on day 5 and day 56, with mean telomere length in each sample expressed relative to a known single-copy gene (the T/S ratio), as detailed by Nettle et al. (2017). We calculated developmental telomere attrition (henceforth ΔTL) as the change in T/S ratio between day 5 and day 56, standardized using the method of Verhulst et al. (2013). A more negative value of ΔTL means a greater degree of developmental telomere attrition as compared to other individuals within our sample, and a positive value means relatively less attrition compared to others in the sample (but does not imply telomere lengthening, Bateson and Nettle 2017). Owing to some failed assays, complete telomere measures were available for 26 birds. The effects of our developmental manipulation on developmental telomere attrition have been previously reported (Nettle, Andrews, Reichert, et al. 2017). Briefly, both Amount of food received and the begging Effort required of nestlings had significant independent effects on ΔTL. Nestlings raised in the food-restricted (Lean) groups and those that were required to spend more time begging (Hard) underwent greater telomere attrition than their siblings receiving ad libitum (Plenty) food and/or rewarded with food at each visit (Easy).

### Training phase

Training began when birds were 264–400 days old and fully grown. Birds were caught from the aviary in groups of 8 and housed in cages (75 × 45 cm and 45 cm high; ca. 18 °C; 40% humidity; 15:9 h light:dark cycle) fitted with 2 wooden perches and 2 water bottles and a water bath, and allowing acoustic and visual contact. The testing apparatus within each cage has been previously described in detail elsewhere (Feenders and Bateson 2013). Briefly, each cage was fitted with an overhead surveillance camera and custom-built operant panel comprising 3 horizontally aligned 4 cm diameter pigeon pecking keys, which could be transilluminated, and a central food hopper connected to an external pellet dispenser delivering 45 mg, grain-based rodent pellets (TestDiet, Richmond, IN). To habituate birds to cages and to socially facilitate their consuming the novel pellets we initially housed birds in pairs (for a minimum of 2 days) with a bowl per bird containing 5 g of pellets and 5 g crumb. On the second and following day(s), we provided 20 g pellets per bird until they were readily consuming pellets, at which point we caged them individually. Thereafter birds received ad libitum food (10 g dry cat biscuits, 5 g chick crumb, 5 g dried insect food, and a slice of fruit) daily between 1230 and 1630 following operant trials (see below), with baths also provided during this period.

Since energy budget is expected to influence risk preference (Caraco et al. 1980; Stephens 1981), we measured the change in body weight of the birds during the course of the experiment as a proxy for energy budget, allowing us to statistically control for individual variation in energy budget (see below). We weighed birds at the time we put them into cages, and likewise when they were removed following the experiment, to measure body weight change (exit minus entry body weight) over the period including training, the risk-sensitivity task and control condition (if performed, see below).

Operant training procedures followed those outlined by Feenders and Bateson (2013). First, birds were exposed to pellet rewards delivered to the hopper (2 pellets every 200 ± 50 s for 80 trials daily) until they reliably consumed the pellets. Next, the birds were autoshaped to peck the centre amber-illuminated key for a food reward, by repeated pairing of the illumination with pellet delivery (15 s illumination, 1 pellet per trial, intertrial interval 100 s, 80 trials daily). We took the speed of autoshaping as a commonly-used measure of learning (Pavlovian conditioning) performance (Markou et al. 2013). The learning speed measure is the (natural log-transformed) number of trials required before the subject first directed an appetitive response (peck) at the stimulus (centre lit key). This measure of learning speed was available for 26 birds (22 with telomere data) owing to the accidental death of one bird (Lean Hard) prior to the experiment, failure of one bird (Lean Easy) to autoshape, and exclusion of learning data from 4 birds (2 Lean Hard, 1 Plenty Hard, 1 Lean Easy) for which we had made minor alterations to the standard autoshaping parameters during the session in which they first pecked (with the aim of hastening their learning). Once a bird started to peck the key, it progressed to a variable number of days of operant training. Each bird received daily sessions of 80 trials until it had pecked on at least 80% of trials in 3 consecutive sessions, or at least 50% of trials in 5 consecutive sessions. When a bird had met this criterion (4–16 days of autoshaping training, mean ± SE 6.5 ± 0.5 days) it progressed to the risk-sensitive foraging task.

### Risk-sensitive foraging task

We used a choice task in which the birds made simultaneous choices between a fixed food reward amount (2 pellets) and a variable food reward amount (1 or 4 pellets with probabilities ^2^/_3_ and ^1^/_3_, respectively) equal in mean reward amount. We selected these reward sizes in order to maximize the coefficient of variation in amount while avoiding empty rewards or satiating birds with very large rewards that may not be completely consumed. Since we were primarily interested in individual differences and hence required all birds to have the same experience, throughout the procedure one pecking key color (green illumination) was assigned to the fixed option, and another color (red illumination) was assigned to the variable option.

Each daily session comprised a maximum of 10 blocks of 12 trials. Sessions began at 0730 and ended after 5 h if a bird had not completed. Each block comprised 6 forced trials followed by 6 choice trials. Within each block, the 6 forced trials were chosen such that there were always 3 of each type (fixed and variable), with 2 variable forced trials yielding a reward of one pellet and one variable forced trial yielding a reward of 4 pellets within each block. Thus, within each block, the forced trials exposed the birds to the programmed distribution of pellets in the variable option. The order in which the 6 forced trials were presented was chosen randomly in each block. At the start of each trial, the central pecking key was illuminated with amber light, and a single peck to this key was required to initiate the trial. On forced trials, following a response to the amber key, the amber light extinguished and either a red or green light appeared on the right or left key (chosen such that red and green were presented equally often on each side across blocks in order to discourage the development of side biases). A single peck to this light initiated the illumination of the hopper light and delivery of the corresponding reward at a rate of one pellet per second. Following the final pellet delivery the intertrial interval of 90 s began. Choice trials were identical to forced trials with the exception that following the initiation peck, both side keys were illuminated (one in red and one in green, with the side randomly chosen). A single peck indicated the bird’s choice and resulted in the keys being extinguished and the corresponding reward delivered. If the variable option was chosen, the number of pellets delivered was chosen randomly according to the designated probabilities (i.e., 1 or 4 pellets with probabilities ^2^/_3_ and ^1^/_3_, respectively) with no constraints. We recorded the birds’ key peck response for either the fixed or variable option in choice trials. Birds were tested 7 days a week and completed between 973–1435 trials, the procedure ending when all birds in each group of 8 had completed at least 1000 trials (excepting one bird, which completed only 973 trials due to time constraints). This large number of trials provided birds with ample opportunity to learn the distribution of pellets associated with the 2 key colors and develop stable risk preferences (see Results for details). As intended, we confirmed that individual birds received a mean reward of 2.00 ± 0.01 (mean ± SE) pellets per choice trial and the proportion of risky choices made did not predict the mean reward per choice trial (LRT = 0.004, *P* = 0.948, Table 1 Model 1). Risk sensitivity data were available on 30 birds.

**Table 1:** Mixed model results

Model	Response variable	Fixed predictor variables	Random effects	LRT	*P*	B (SE)	*n*	AICc
1	Mean reward per choice trial	Proportion of risky choices	Family	0.004	0.948	0.005 (0.08)	30	
2	ΔTL	Starting TL	Family	1.86	0.173	−0.16 (0.10)	26	
3	Cage entry body weight	ΔTL	Family	8.72	0.003	16.83 (4.71)	26	
4	BWC	ΔTL	Family	5.19	0.023	−11.75 (4.38)	26	
5	Variable reward chosen	Scale (ΔTL)	Family/Bird	7.51	0.006	0.17 (0.07)	26	10151.26
		Scale (BWC)		10.25	0.001	0.20 (0.08)		
		Scale (ΔTL) × scale (BWC)		1.59	0.207	0.09 (0.07)		
6	Variable reward chosen	Scale (Juvenile TL)	Family/Bird	0.21	0.645	0.03 (0.08)	26	10160.06
		Scale (BWC)		4.77	0.029	0.19 (0.08)		
		Scale (Juvenile TL) × scale (BWC)		0.09	0.768	−0.02 (0.06)		
7	Variable reward chosen	Scale (BWC)	Family/Bird	3.22	0.073	0.17 (0.08)	26	10159.96
		Amount:Plenty^a^		0.82	0.366	−0.25 (0.22)		
		Effort:Hard^b^		1.11	0.292	−0.27 (0.21)		
		Amount × Effort		0.47	0.492	0.21 (0.30)		
8	Variable reward chosen	Scale (BWC)	Family/Bird	4.64	0.031	0.18 (0.08)	26	10156.35
9	Variable reward chosen	Scale (ΔTL)	Family/Bird	1.90	0.169	0.13 (0.08)	26	10159.10
10	ln(Learning speed)	ΔTL	Family	1.99	0.159	−2.56 (1.86)	22	
11	ln(Learning speed)	BWC	Family	0.03	0.853	0.01 (0.05)	26	
12	ln(Learning speed)	Juvenile TL	Family	0.32	0.573	0.57 (1.05)	22	
13	ln(Learning speed)	Amount:Plenty^a^	Family	0.91	0.341	1.45 (0.99)	26	
		Effort:Hard^b^		0.03	0.865	1.11 (1.11)		
		Amount × Effort		1.61	0.204	−1.73 (1.45)		
14	Variable reward chosen	Scale (Learning speed)	Family/Bird	0.45	0.500	−0.06 (0.09)	26	

Scaled continuous fixed predictors are used in models 5–9 for the purposes of comparing effect sizes. Starting TL is telomere length measured by T/S ratio at day 5. BWC is Body Weight Change. ΔTL is developmental telomere attrition from day 5 to day 56. Juvenile TL is telomere length measured by T/S ratio at day 56. Learning speed is the number of autoshaping trials until the bird first pecked the lit key.

^a^Reference group for Plenty is Lean. ^b^Reference group for Hard is Easy.

### Control conditions

In order to verify that the birds were able to learn and respond to the variable reward distribution, we conducted 2 additional conditions with a subset of 12 birds (from 4 natal families). Once the first 12 birds (4 Plenty Hard, 3 Plenty Easy, 3 Lean Easy, 2 Lean Hard) had completed the risk-sensitivity condition described above, we randomly allocated 6 birds to each of 2 new conditions. In the first condition (Fixed Halved), we reduced the reward amount of the fixed option to 1 pellet rather than the 2 pellets previously. We predicted that birds in this condition should prefer the variable option (i.e., be strongly risk prone) since this now yielded a higher mean amount (2 pellets in variable vs. 1 pellet in fixed). In the second condition (Risky Win Halved), we reduced the reward amount in the variable option to 1 pellet or 2 pellets (with probabilities remaining ^2^/_3_ and ^1^/_3_, respectively). We predicted that birds in this condition should prefer the fixed option (i.e., be strongly risk averse) since this now yielded a higher mean amount (2 pellets in fixed vs. 1.3 pellets in variable). Birds completed 696–2613 trials of the additional condition.

### Statistical analysis

The raw data and R script are archived in the Zenodo repository (doi: 10.5281/zenodo.848211). Statistical analyses were conducted in R v.3.4.1 (“R Development Core Team” 2011) using the base statistical procedures and “nlme” (Pinheiro et al. 2015) and “lme4” packages (Bates et al. 2015). We used generalized linear mixed models incorporating random intercepts for natal family and, where appropriate due to repeated measures, individual identity. Error distribution was Gaussian (identity link) where mean reward per trial, ΔTL, entry body weight, body weight change or leaning speed was the outcome, or binomial (logit link) where the outcome was binary choice between the variable or fixed reward option. The fixed effects included in each model are described in the relevant results section and in Table 1. Maximum-likelihood estimation was employed throughout. Significance testing was carried out by the likelihood ratio test (LRT), which compares the change in deviance when a term is excluded from the model with the χ^2^ distribution with 1 degree of freedom. We assumed a criterion for significance of *P* < 0.05.

To examine risk preference, we treated the first 400 trials of the risk-sensitive foraging task as the period of discrimination training, since our control conditions confirmed this to be sufficient experience for birds to learn a discrimination based on fixed or variable reward amounts with unequal mean amounts (see Results for details). Therefore, we regarded the choice trials between the 400^th^ and 1000^th^ trial of either type (forced or choice) as yielding a stable measure of risk preference for each bird. Our main model included ΔTL (developmental telomere attrition between day 5 and day 56) and body weight change (during the full period the bird was caged for this experiment) as predictors of choice of fixed or variable reward amounts. In subsequent analyses we also explored the effects of replacing ΔTL with juvenile telomere length (i.e., day 56 T/S ratio) or developmental manipulation (see Results for details). Starting telomere length (day 5 T/S ratio) did not significantly predict ΔTL in these birds (see Results for details, Table 1 Model 2; LRT = 1.86, *P* = 0.173) and therefore was not included as a covariate in models with ΔTL as predictor. We compared models using the R package “AICcmodavg” (Mazerollle 2016) and a modified version of Akaiki’s Information Criterion (AICc) recommended for small sample sizes (Symonds and Moussalli 2010). For the visual representation of the main results (Figure 2), we calculated overall risk preference for each bird as the proportion of choices for the variable reward made in choice trials (between the 400^th^ and 1000^th^ trial on the risk-sensitive foraging task). We then plotted the association between overall risk preference and ΔTL after controlling for body weight change, and between overall risk preference and body weight change after controlling for ΔTL, using the R package “visreg” (Breheny and Burchett 2016).

## RESULTS

### Body weight change

Birds that underwent greater developmental telomere attrition (i.e., more negative ΔTL) were initially lighter at cage entry for the current experiment (LRT = 8.72, *P* = 0.003, Table 1 Model 3), but lost less body weight (i.e., more positive values of body weight change) during the experiment (LRT = 5.19, *P* = 0.023, Table 1 Model 4).

### Control conditions

**Figure 1:**
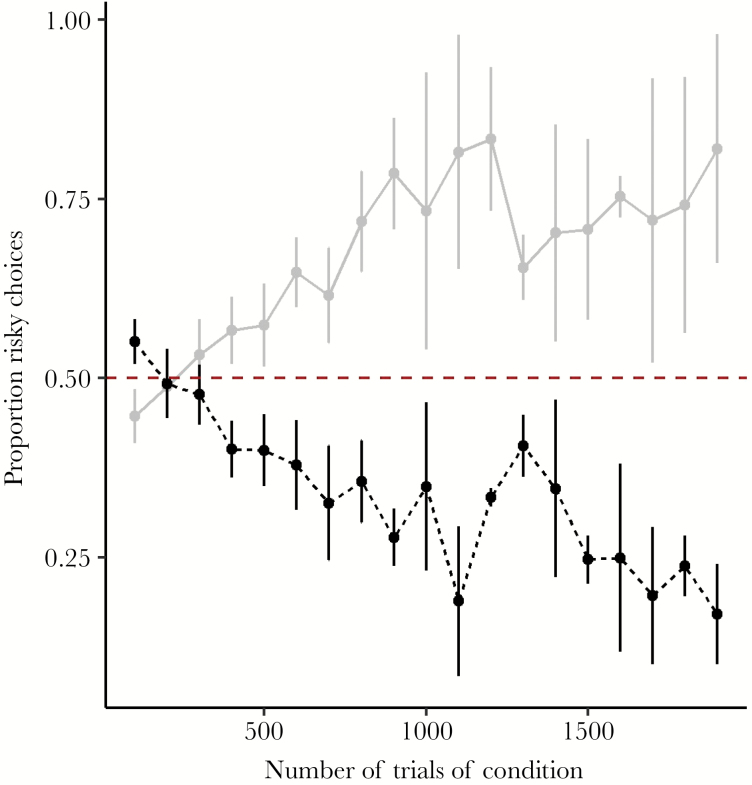
Mean (±SE) proportion (per 100 trials) of choices for the variable (risky) reward in choice trials during control conditions. Fixed Halved condition (gray solid line, *n* = 6): fixed payoff = 1 pellet, risky payoff = 1 pellet with probability ^2^/_3_ or 4 pellets with probability one third. Risky Win Halved condition (black dashed line, *n* = 6): fixed payoff = 2 pellets, risky payoff = 1 pellet with probability two thirds or 2 pellets with probability one third. Mean proportion of risky choices are calculated over each 100 trials (fixed or choice). The red dashed line indicates indifference to risk. Data are shown up to 2000 trials since few birds completed more.

Figure 1 shows that mean risk preference for birds in the Fixed Halved condition diverged from that of birds in the Risky Win Halved condition over increasing numbers of trials; birds in the Fixed Halved condition became increasingly risk prone whereas those in the Risky Win Halved condition became increasingly risk averse. As expected, overall mean risk preference across all choice trials performed by birds in the Fixed Halved condition was significantly risk prone (one-sample 1-tailed *t*-test against µ = 0.5, *t*_5_ = 2.18, *P* = 0.040) while overall mean risk preference for birds in the Risky Win Halved condition was significantly risk averse (one-sample 1-tailed *t*-test against µ = 0.5, *t*_5_ = −3.24, *P* = 0.011).

### Risk sensitivity

**Figure 2:**
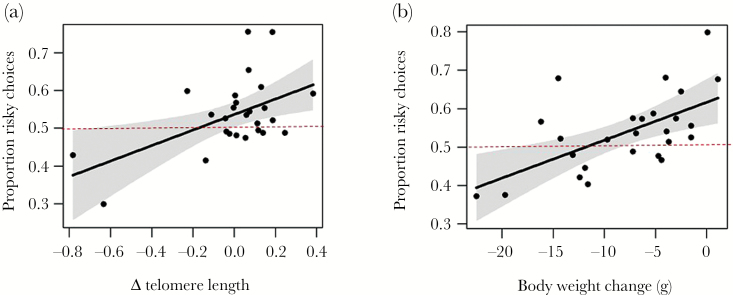
Predictors of risk preference. Proportion of choices for the variable (risky) reward over the fixed reward in choice trials and (a) developmental telomere attrition (a more negative value of Δ telomere length means greater attrition); (b) body weight change (a more negative value means greater weight loss). The figure shows one data point per bird representing the proportion of choices for the variable reward made in choice trials between the 400^th^ and 1000^th^ trial on the risk-sensitive foraging task, after adjusting for the other predictor in the model. The red dashed line indicates indifference to risk. The solid line is the line of best fit from a simple linear regression model, with 95% CIs shaded in gray.

To ascertain whether adult risk preference was predicted by developmental telomere attrition, we modeled the choice of fixed or variable reward amount with ΔTL as predictor, also including body weight change as a predictor (Table 1 Model 5). Birds that had undergone less developmental telomere attrition as nestlings and those that lost less body weight during the experiment made more risky choices (Figure 2, Table 1 Model 5, ΔTL LRT = 7.51, *P* = 0.006; Body weight change LRT = 10.25, *P* = 0.001), but there was no interaction between ΔTL and body weight change (Table 1 Model 5, LRT =1.59, *P* = 0.207). The effect of ΔTL on risk preference was slightly weaker than that of body weight change (scaled parameter estimates β ± SE: 0.17 ± 0.07 and 0.20 ± 0.08, respectively; Table 1 Model 5). This model was the best-fitting (lowest AICc), and the evidence ratio showed this to be 12.8 times more likely to be the best-approximating model than a model with body weight change as sole predictor (Table 1 Model 8). We then modeled choice of the variable or fixed reward but replaced ΔTL by juvenile telomere length (day 56 T/S ratio) as predictor, retaining body weight change as covariate. Telomere length did not significantly predict birds’ choices for the variable reward (Table 1 Model 6, LRT = 0.21, *P* = 0.645), although the direction of the effect was the same as that of ΔTL (i.e., shorter telomeres associated with fewer risky choices). The evidence ratio showed this model (Table 1 Model 6) to be 81.5 times less likely to be the best-approximating model than the previous model with ΔTL as predictor (Table 1 Model 5). Last, we examined the effect of our developmental manipulations themselves on risk preference by replacing ΔTL by the Amount and Effort treatments as fixed factors in the model along with their interaction, retaining body weight change as covariate (continuing to restrict analysis to birds with telomere data to allow comparison). Neither Amount, Effort nor their interaction significantly predicted choice for the variable reward (Table 1 Model 7).

### Speed of learning

Learning speed, as measured by the number of autoshaping trials until a bird first responded by pecking the lit key, was not predicted by developmental telomere attrition, body weight change, juvenile telomere length, or developmental treatment (Table 1 Models 10–13). Risk preference was not predicted by learning speed (Table 1 Model 14).

## DISCUSSION

Our aim was to test the prediction that greater developmental telomere attrition, a measure of biological ageing, is associated with more risk-prone foraging in starlings choosing between fixed and variable-amount food rewards with equal mean. We found that developmental telomere attrition as well as body weight change (a proxy for energy budget) independently predicted foraging risk preference, however the effect of developmental telomere attrition was in the opposite direction to our prediction. Specifically, birds that had previously undergone greater developmental telomere attrition as nestlings, and those that lost more body weight during the risk-sensitive foraging experiment as adults, had a stronger preference for fixed over variable reward amounts (i.e., were more risk averse) than those that had undergone less developmental telomere attrition or lost less weight. As we had expected, developmental telomere attrition was a stronger predictor of adult risk preference than either juvenile telomere length or the developmental manipulation per se.

We have shown here for the first time that a marker of biological ageing, developmental telomere attrition, predicts risk preference in a nonhuman animal. In our dataset, the association between developmental telomere attrition and risk preference was only slightly weaker than that between body weight change, a proxy measure of energy budget, and risk preference. However, the direction of our result runs contrary to the prediction based on McNamara et al.’s (1991; see also Merad and McNamara 1994) theoretical model which showed that increasing background mortality (as expected under faster biological ageing) promotes risk prone foraging in animals which have sufficient reserves for their starvation risk to be low. The starvation risk of our birds was low because they had access to daily ad libitum food and maintained healthy body weights, and the mean food reward amount was positive as required for this model prediction. Thus, it is reasonable to assume our birds were within the region of the model space in which raising mortality is predicted to increase risk proneness. Although our birds were still relatively young at the time of testing (starlings have a maximum longevity of 22.9 years; AnAge database, Tacutu et al. 2013), it is likely that the variation in mortality associated with developmental telomere shortening in our starling cohort was biologically meaningful at the age at which we measured risk preference: Although data linking developmental telomere attrition to mortality rates is currently unavailable for starlings, a study of wild jackdaws (*Corvus monedula*) found that nestling telomere shortening had a strong effect on postfledging survival (Boonekamp et al. 2014). It is therefore unclear why our results diverge from the model prediction.

Although our findings contradict the specific predictions of McNamara et al.’s (1991) model, they are nevertheless compatible with the broader reasoning that biological ageing could influence risk preference by reducing residual reproductive value. A change in residual reproductive value should alter the trade-off between survival and reproduction (Kirkwood and Rose 1991; Stearns 1992; Monaghan 2008) and hence the function linking food rewards to fitness gain. Our finding also fits with theoretical models showing that an animal’s time horizon could affect the difference in expected fitness between certain or risky feeding options, and thus whether risk-proneness or risk-aversion be adaptive (Lim et al. 2015). One possibility is that individuals with faster biological ageing, and hence shorter life expectancy, have less time available in which to recover from a possible series of losses in order to successfully reproduce, and thus are more averse to risk. Another recent evolutionary model showed that risk aversion is influenced by stochastic properties of the environment that affect reproductive success (Zhang et al. 2014). Such environmental variation (e.g., natural disasters) could have a relatively greater influence on the lifetime reproductive success of shorter-lived individuals than longer-lived ones, leading more rapidly ageing individuals to show greater risk aversion. Further formal theoretical exploration of the link between ageing-related mortality or lowered residual reproductive value and risk preference would be informative.

To our knowledge, ours is the first study showing ageing-related variation in risk preference in a nonhuman animal. In rats, chronologically older animals performed comparably to young on average in a risky choice task, although there was greater between-individual variation in risk preference among older rats (Gilbert et al. 2012). In humans, the prevailing finding has been for greater risk aversion with chronological ageing (Harbaugh et al. 2002; Deakin et al. 2004; Dohmen and Falk 2010; Mohr et al. 2010; Boyle et al. 2011; Rutledge et al. 2016; although see Cavanagh et al. 2012; Pachur et al. 2017), a pattern similar to that in our starlings in respect of our biomarker of biological ageing. Increased risk aversion was also observed in human experimental participants primed to perceive greater extrinsic mortality risk, and therefore potentially perceive lowered life expectancy (Pepper et al. 2017). However, a single study in humans examining the association between risk preference and telomeres found greater risk proneness in a stock investment task to be associated with shorter telomere length (Yim et al. 2016). In contrast, we found developmental telomere attrition to be a better predictor of starlings’ risk preference than juvenile telomere length, the latter effect being in the same direction as that of developmental telomere attrition but not statistically significant. Telomere attrition rather than absolute telomere length may be the better predictor of longevity (Boonekamp et al. 2014) and hence biological ageing. Our finding aligns with mounting evidence from birds that developmental telomere attrition is a stronger predictor of adult phenotypic outcomes than a single cross-sectional measurement of adult telomere length (reviewed in Andrews, Nettle, Larriva, et al. 2017). Since we measured telomere length in juveniles (day 56) and risk preference in adulthood (>day 260), in future studies it would be useful to acquire telomere length measures contemporaneously with behavioral measures of decision-making for closer comparison to the data on humans. However, we note that developmental telomere attrition predicted adult telomere length in 2 separate cohorts of starlings (Bateson, Brilot, et al. 2015; Andrews, Nettle, Larriva, et al. 2017) as well as in jackdaws (Boonekamp et al. 2014).

Our finding of greater risk aversion among birds that had lost more body weight is inconsistent with original formulations of the energy budget rule, which predicts animals with negative energy balance and hence losing body weight should be risk prone for reward amount (Caraco et al. 1980; Stephens 1981). We do not however view this as contradictory to current risk sensitivity theory on dual grounds. First, more recent theoretical formulations of risk sensitivity theory predict risk aversion on a negative energy budget only under certain circumstances (Kacelnik and El Mouden, 2013; Lim et al., 2015). Second, we did not manipulate the energy budgets of our starlings. Instead, the birds had access to ad libitum food during periods of each day, thus adjustments in body weight were unlikely to be due to insufficient opportunity for calorific intake, as was the case in Caraco’s (1980, 1981, 1990) seminal empirical demonstrations of the energy budget rule. Adjustments in body weight in our starlings may have been strategic (e.g., relating to altered social interactions or flight requirements when caged; Witter and Swaddle 1995; or to altered perceived prediation risk; Rogers 2015) rather than due to energetic constraint. Greater strategic weight loss when individually caged might imply greater perceived food security (Nettle, Andrews, and Bateson 2017), which seems compatible with a broader interpretation of the energy budget rule: birds that potentially perceived their energy balance as least threatened showed greatest aversion to risk in our study.

We did not find a direct effect of early developmental experience, in the form of our developmental manipulation, on later risk preferences. This was somewhat surprising for 2 reasons. First, prior conditions experienced and expectations learned have previously been shown to influence animals’ decision-making in the context of risk (Marsh and Kacelnik 2002; Bacon et al. 2010; Kacelnik and El Mouden 2013). Second, sensitivities to reward gain and loss may underlie risk-sensitive decisions (Eppinger et al. 2011), and we have previously found evidence in this starling cohort that our developmental manipulation affected sensitivity to shifts in reward magnitude (Neville et al. 2017). Nonetheless, comparable to our present finding, rats reared in impoverished and enriched conditions did not differ in risk preference (Kirkpatrick et al. 2014). That telomere attrition rather than developmental manipulation best predicted the phenotypic outcome is in agreement with what would be expected if telomere attrition serves as an integrative measure of the combined experiences during development (not limited only to our manipulation) and individual variation in sensitivity to those experiences (Bateson 2016). We have previously found telomere attrition to be the better predictor of a range of phenotypic outcomes in starlings compared to developmental manipulations themselves (Bateson, Brilot, et al. 2015; Nettle, Andrews, et al. 2015; Andrews, Nettle, Larriva, et al. 2017).

Decision-making under risk involves neurobiological substrates which partially overlap those underpinning decisions involving time delay to reward in both rats (Kirkpatrick et al. 2014) and humans (Mohr et al. 2010), suggesting that the same processes play a role in both risky and impulsive choices. In a separate study of a different cohort of starlings, we found that birds that underwent greater developmental telomere attrition made more impulsive foraging decisions, preferring smaller but more immediate food rewards over larger delayed rewards (Bateson, Brilot, et al. 2015; see also Nettle, Andrews, et al. 2015). Delay-to-reward may be viewed as a source of uncertainty, since interruptions in the meantime (e.g., arrival of a predator or competitor) could lead to variation in the actual reward obtained. Hence both delayed reward or variable reward amounts represent uncertain, “risky” options. Impulsivity and risky choice are related in pigeons (Laude et al. 2014) and rats (Kirkpatrick et al. 2014) as well as humans (Alessi and Petry 2003; Baumann and Odum 2012); in these cases, greater impulsivity was associated with greater risk proneness. Our starling studies taken together indirectly imply the reverse association, that is greater developmental telomere attrition was predictive of greater impulsivity but also of greater *aversion* to risk. In another prior study on previous starling cohorts, we found that birds undergoing greater developmental telomere attrition had an attenuated hypothalamic-pituitary-adrenal (HPA) axis glucocorticoid hormone stress response (Andrews, Nettle, Larriva, et al. 2017). Within neuroeconomics, evidence is emerging for a role for glucocorticoids as mediators of human financial risk-taking (Coates et al. 2010; Kandasamy et al. 2014). Experimentally sustained elevation in cortisol leads to greater risk aversion in humans (Kandasamy et al. 2014), the opposite pattern to that indirectly implied by our starling studies in combination. These apparent species differences might be accounted for by methodological differences (e.g., experiential vs. descriptive presentation of choice outcomes). Our combined findings in starlings nevertheless add to those in humans and rodents suggesting that attitudes towards uncertainty may vary with ageing.

An alternative to the proposed adaptive rationale for altered risk preference with biological ageing discussed above is that altered decision-making could be a product of ageing-related cognitive impairment (as in humans, Boyle et al. 2011; Pachur et al. 2017). However, we think it unlikely that differences in ability to learn the task contingencies underlies the effect of biological ageing on risk preference in our starlings. Our birds were still relatively young (264–400 days in a species with a potential lifespan of >20 years; AnAge database, Tacutu et al. 2013), and in the present study, we found no association of the speed of learning with developmental telomere attrition or with risk preference, thus cognitive senescence is unlikely to account for altered risk preference in our study. Additionally, in previous studies on separate cohorts of starlings, we found only limited associations between developmental telomere attrition and learning ability (Bateson, Brilot, et al. 2015; Nettle, Andrews, et al. 2015). We note however that greater developmental telomere attrition was associated with slower autoshaping performance in one of these cohorts (Nettle, Andrews, et al. 2015), but also that this measure may reflect differences in neophobia rather than purely cognitive ability (Feenders and Bateson 2013). Similarly, risk preference among aged rats was not associated with performance in a spatial learning task (Gilbert et al. 2012). It must in addition be emphasized that our result is essentially correlational—telomere loss, as a biomarker of biological ageing, predicted risk-taking behavior, yet the link is not necessarily causally mechanistic (for discussion of causal links between telomere dynamics and behavior, see Bateson and Nettle 2018). Telomere attrition may not directly modulate risk-taking, but rather, biological ageing (as measured by telomere attrition) may alter some unknown variable (for example, corticosterone, as discussed above) which modulates risky decision-making.

In conclusion, we have reported the first evidence that biological ageing, as measured by developmental telomere attrition, predicts risky decision-making in any species. Our study contributes novel evidence that an aspect of an individual’s state besides energy budget predicts foraging risk preference, a finding which should inspire broadening of theoretical models of risk-sensitive foraging. More generally our results fit with substantial evidence that biological ageing can have profound effects on adult behavioral phenotypes, raising questions about the biological embedding of early-life adversities which accelerate ageing.

## SUPPLEMENTARY MATERIAL

Supplementary data are available at *Behavioral Ecology* online.

Supplementary Material

## FUNDING

This work was supported by the Biotechnology and Biological Sciences Research Council (grants BB/J016446/1 and BB/J016292/1) and European Research Council (AdG 666669).

## 

Data Accessibility: Analyses reported in this article can be reproduced using the data provided by Andrews, Nettle, Reichert, et al. (2017).
