# Supplementary material for: A marker of biological ageing predicts adult risk preference in European starlings, Sturnus vulgaris
Source: Behav Ecol. 2018 Feb 24;29(3):589–97. doi: 10.1093/beheco/ary009 (PMC5946890; doi:10.1093/beheco/ary009)
Supplement: Supplementary Material [file ary009_suppl_supplementary_materials.docx]

## Supplementary material

### Ethical notes

Removal of nestlings in the field was authorized by Natural England and carried out with the permission of landowners, with nest disturbance minimised. There was no mortality among our hand-reared nestlings and all but one gained weight between removal and the following evening, suggesting rapid recovery from transport and acceptance of hand feeding. The manipulation was intended to increase developmental stress in the Lean and Hard groups. However, we designed the feeding protocol to produce growth curves following closely those observed among nestlings in the wild with either larger (Lean Hard developmental treatment group) or smaller brood competitors (Plenty Easy developmental treatment group) (Nettle et al. 2015. *Proc. Biol. Sci.* 282, 20141610). Thus, the level of developmental stress was likely to have been within the naturally experienced range. The manipulation was also likely in many cases to reduce a nestling's developmental stress compared to that which it would have experienced in its natal nest. Aviary-housed birds were provided with environmental enrichments consisting of foraging substrate, water baths, multilevel rope perches, and suspended cardboard boxes as cover. The blood sampling procedure was refined by using as small a needle (25 gauge) and sample volume as possible. Combined blood sample volume was well below the UK legal limits for the percentage of total blood volume. After sample collection, blood flow was stemmed under careful observation by application of firm pressure using a cotton wool swab. Birds were returned to their aviary within a maximum of 44 days of removal for risk-sensitivity measurement. Following this study, birds were kept for further experiments at Newcastle University.
